# Stop Using the Eccentric Exercises as the Gold Standard Treatment for the Management of Lateral Elbow Tendinopathy

**DOI:** 10.3390/jcm11051325

**Published:** 2022-02-28

**Authors:** Dimitrios Stasinopoulos

**Affiliations:** Laboratory of Neuromuscular & Cardiovascular Study of Motion (LANECASM), Department of Physiotherapy, Faculty of Health and Caring Sciences, University of West Attica, 12243 Athens, Greece; dstasinopoulos@uniwa.gr

The most common tendinopathy in the elbow area is the Lateral elbow tendinopathy (LET). The diagnosis of LET is simple and quick. The majority of clinicians advocated a conservative approach. Physiotherapy is usually provided. Manual techniques, external support, soft tissue manipulation, physical agents and heavy-slow resistance exercise are recommended physiotherapy approaches for the management of LET.

The most common physical therapy approach for LET is a progressive loading supervised or in clinical placement exercise programme [1]. Progressive eccentric training has shown good clinical results in LET patients [2,3,4]. Such an exercise training is used as the first treatment option for LET patients [5]. However, many patients with LET do not respond to eccentric exercises alone [1,6,7]. Thus, eccentric exercises are combined with static stretching exercises for extensor carpi radialis brevis (ECRB) with positive results [8,9,10,11]. Stretching may make the tendon more resistant to strain or strengthen it increasing the range of motion of the relevant joint [12,13]. Moreover, stretching contribute to the orientation of the new collagen fibres with a “lengthening” of the muscle-tendon unit [14].

The management of LET is changing, and in our days eccentric training is not the only exercise option [1]. Few studies on progressive eccentric training have been published in the last decade [6]. Physicians should consider concentric-eccentric training alongside or instead of eccentric training for the rehabilitation of LET [15].

Martinez-Silvestrini et al. [16] stated that LET patients require isometric contraction because LET is often related to forceful grip activities. More research is needed to draw conclusions about the effectiveness of isometric training in tendinopathy since conflicting results have been found in terms of immediate and short-term pain relief [17,18,19].

It was hypothesized that static stretching exercises and the simultaneous use of isometric and isotonic contractions will decrease the pain and improve the function in LET patients. The results obtained from a randomized clinical trial showed that the eccentric—concentric training combined with isometric contraction produced the largest effect at the end of treatment and at the follow up [20].

The exercise training in LET should include progressive strengthening exercises not only for ECRB but also for supinator, rotator cuff and scapular muscles [21,22] Using supinator, rotator cuff and scapular muscles progressive strengthening loading, LET patients will carry out painless activities such as gripping, increasing the function of the arm.

Moreover, reduced proprioception have also been noticed in LET patients [23]. The reduced proprioception is usually ignored by clinicians in the treatment of LET and thus the symptoms may persist for a long period of time and recurrence is common. If physiotherapists use approaches to improve reduced proprioception, the results will be effective earlier [24].

In addition, tendon neuroplastic training (TNT) is recommended to address the central nervous system component of tendinopathy [25,26,27]. The other recommended conservative treatment approaches for tendinopathy such LET do not address the motor control deficits [27].

Finally, motor and sensory system deficits are common in the non-injured extremity of patients with unilateral LET [28]. This suggests that specific training of the contralateral extremity may also provide additional benefits to the affected extremity through cross education [29]. Unfortunately, a pilot study did not support the previous [30].

According to above mentioned topics, it is time to stop using only eccentric training for LET. The LET therapy should be based on a progressive loading of the whole upper limb as a kinetic chain. However, the optimal protocol of such an exercise loading requires to be investigated.

The research evidence to support the effectiveness of the physiotherapy approaches mentioned in the first paragraph of this editorial as monotherapy for the management of LET is conflicting. All these recommended physical therapy modalities should not be substitute but instead added to an exercise program. More research is required to discover which treatment approach, if exists a treatment approach, combined with progressive exercise loading will provide the best results in LET patients.

Overall, I trust that the issues raised in this editorial might help to interpret the findings of the present clinical practice. It is authors’ intention to generate questions about the optimal treatment approach for the therapy of LET.
